# Unidirectional Operation of p-GaN Gate AlGaN/GaN Heterojunction FET Using Rectifying Drain Electrode

**DOI:** 10.3390/mi12030291

**Published:** 2021-03-10

**Authors:** Tae-Hyeon Kim, Won-Ho Jang, Jun-Hyeok Yim, Ho-Young Cha

**Affiliations:** School of Electrical and Electronic Engineering, Hongik University, 94 Wausan-ro, Mapo-gu, Seoul 04066, Korea; jxajxa@g.hongik.ac.kr (T.-H.K.); jwh8904@mail.hongik.ac.kr (W.-H.J.); jhgjhg4@g.hongik.ac.kr (J.-H.Y.)

**Keywords:** AlGaN/GaN heterojunction, p-GaN gate, unidirectional operation, rectifying electrode

## Abstract

In this study, we proposed a rectifying drain electrode that was embedded in a p-GaN gate AlGaN/GaN heterojunction field-effect transistor to achieve the unidirectional switching characteristics, without the need for a separate reverse blocking device or an additional process step. The rectifying drain electrode was implemented while using an embedded p-GaN gating electrode that was placed in front of the ohmic drain electrode. The embedded p-GaN gating electrode and the ohmic drain electrode are electrically shorted to each other. The concept was validated by technology computer aided design (TCAD) simulation along with an equivalent circuit, and the proposed device was demonstrated experimentally. The fabricated device exhibited the unidirectional characteristics successfully, with a threshold voltage of ~2 V, a maximum current density of ~100 mA/mm, and a forward drain turn-on voltage of ~2 V.

## 1. Introduction

AlGaN/GaN heterojunction field-effect transistors (HFETs) have been extensively studied for high-efficiency power switching and high-frequency applications owing to their properties, such as wide energy bandgap, high critical electric field, and two-dimensional electron gas (2DEG) channels with high electron mobility and electron density [1,2,3,4,5,6,7]. While the power switching devices must be operated in a normally-off mode, conventional AlGaN/GaN HFETs exhibit normally-on characteristics. A widely adopted device structure for the normally-off mode is a p-GaN gate AlGaN/GaN HFET, where the gate region has a p-GaN layer to deplete the area underneath the AlGaN/GaN channel [4,8,9,10,11,12,13]. Such device types have been successfully commercialized and they are currently used in various power modules for different electronic devices, such as fast chargers, switching mode power supplies, and lighting drivers. Some applications of switching devices are to prevent reverse conduction in order to protect the circuit, so-called unidirectional switching characteristics. A reverse blocking device or circuit must be added to the switching device to achieve unidirectional characteristics, which enlarges the chip size and increases the manufacturing cost. Some studies have reported the unidirectional operation of GaN devices without adding extra components [14,15,16,17,18]. In this study, we proposed a unidirectional switching device that is based on a normally-off p-GaN gate AlGaN/GaN HFET in which a drain electrode consisted of a rectifying gating electrode and an ohmic electrode. The proposed device requires no separate blocking device or additional manufacturing costs.

## 2. Device Structure and TCAD Simulation

### 2.1. Simulation Details

**Figure 1:**
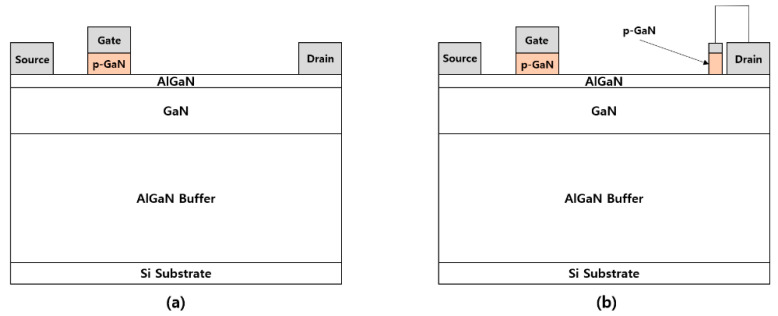
Cross-sectional schematics of (**a**) p-GaN gate AlGaN/GaN heterojunction field-effect transistor (HFET) and (**b**) unidirectional HFET.

The epitaxial structure used for device simulation consists of a 70 nm p-GaN layer with a p-type doping concentration of 3 × 10^17^ cm^−3^, a 15 nm unintentionally-doped Al_0.2_Ga_0.8_N barrier layer with an n-type doping concentration of 1 × 10^16^ cm^−3^, a 35 nm unintentionally-doped GaN channel layer with an n-type doping concentration of 1 × 10^16^ cm^−3^, and a 1.95 μm Al_0.05_Ga_0.95_N buffer layer. Figure 1a,b demonstrate the cross-sectional schematics of a conventional p-GaN gate AlGaN/GaN HFET and a proposed unidirectional device, respectively, with a gate length of 2 μm for both of the structures. The length of the p-GaN drain region was 1 μm for the unidirectional device, which was separated from the drain electrode by 0.5 μm.

**Figure 2:**
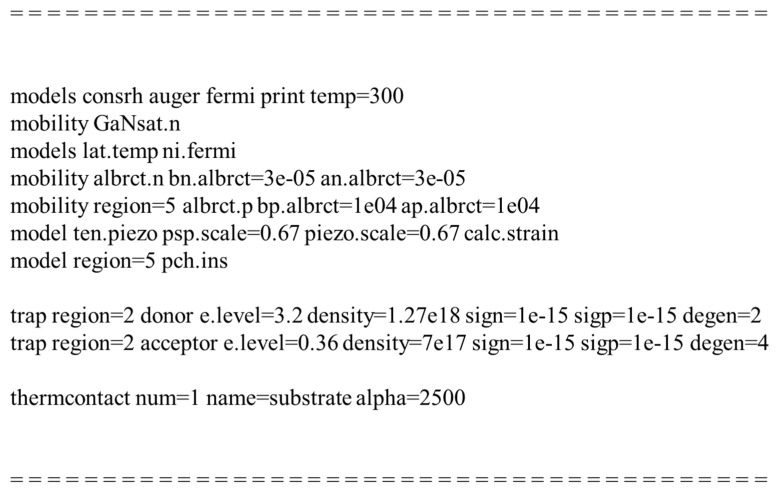
Physical models and parameters used in simulation code.

The simulations were carried out using SILVACO ATLAS (Silvaco, Silicon Valley, CA, USA). Figure 2 shows the models used in the simulation code, which was adopted from an example file provided by SILVACO (ganfetex07.in). A detailed explanation of the simulation models can be found in ref [19], which includes a polarization model, a temperature dependent low field mobility model, a nitride specific high field dependent mobility model, a lattice heating model, and a trap model.

### 2.2. Simulation Result and Disscussion

**Figure 3:**
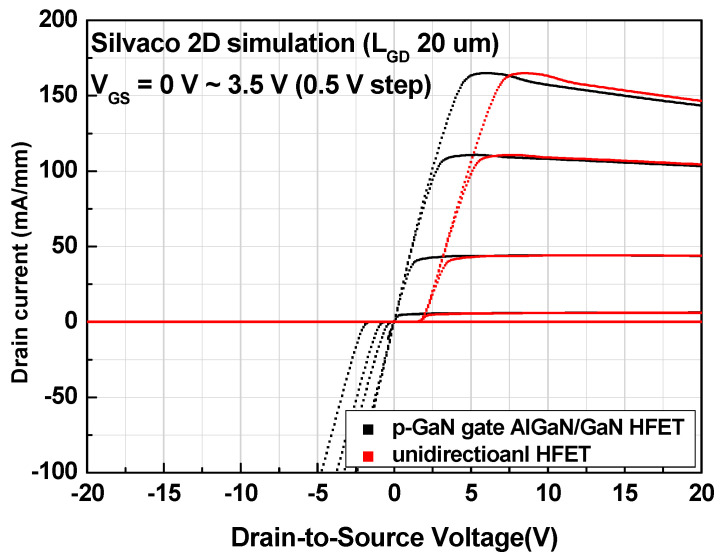
Forward and reverse characteristics of p-GaN gate AlGaN/GaN HFET (black lines) and unidirectional HFET (red lines).

**Figure 4:**
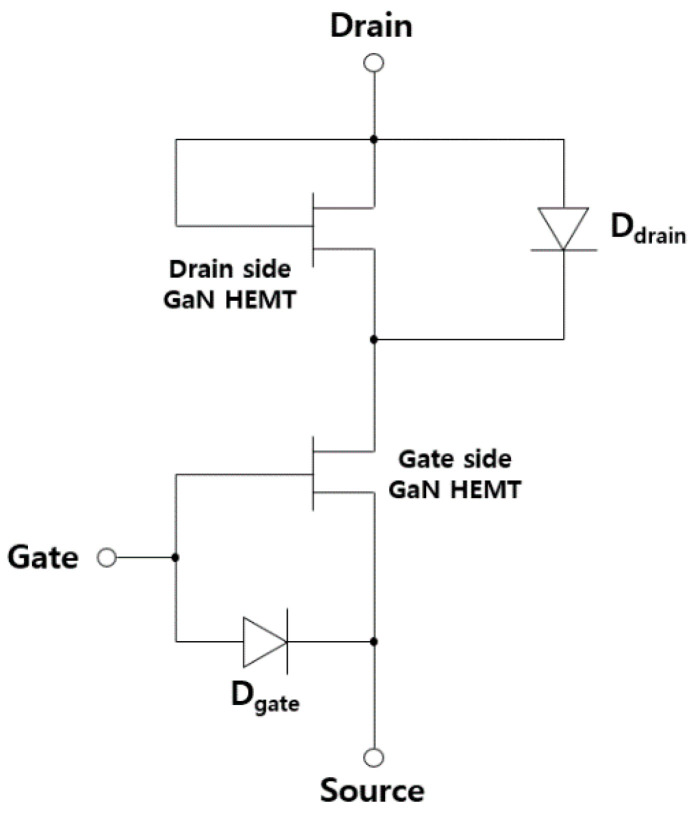
Equivalent circuit of unidirectional HFET.

**Figure 5:**
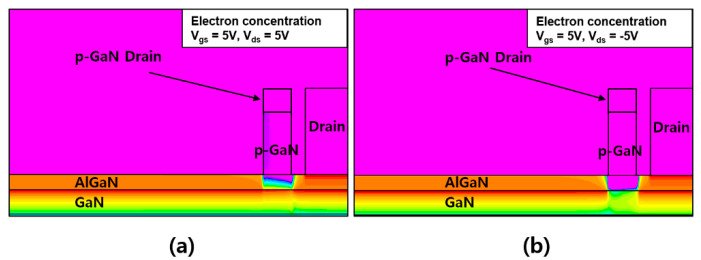
Electron concentration under the p-GaN drain region at (**a**) *V*_gs_ = 5 V and *V*_ds_ = 5 V and (**b**) *V*_gs_ = 5 V and *V*_ds_ = −5 V. Two electrodes (p-GaN drain and drain electrodes) are shorted electrically to each other in the simulation.

Figure 3 compares the simulation results of the forward and reverse characteristics for two different structures. The conventional device exhibited a typical normally-off operation with reverse conduction characteristics, whereas the proposed structure exhibited the same normally-off operation with reverse blocking characteristics. The threshold voltage was 1.8 V for both devices, which was determined by the p-GaN gate electrode. A positive shift in the forward drain turn-on characteristics was observed for the proposed unidirectional device, which is the same as the gate threshold voltage of the device. The positive shift and reverse blocking characteristics can be explained while using the equivalent circuit that is shown in Figure 4. The p-GaN gate can be represented by a gate electrode of the HFET in conjunction with a PN heterojunction diode. When the p-GaN gate voltage exceeds the threshold voltage (1.8 V), the 2DEG channel is formed between the AlGaN barrier layer and GaN channel layer, creating a conduction path between the source and drain. As the p-GaN gate voltage becomes higher than the forward turn-on voltage of the p-GaN/AlGaN/GaN heterojunction diode, the current flows from the p-GaN gate to the source. On the drain side, the p-GaN region acts as a “gate” electrode, which is electrically shorted to the ohmic electrode. Therefore, the current can flow from the ohmic drain electrode to the source electrode by creating the 2DEG channel under the p-GaN region, as the drain voltage becomes higher than the gate threshold voltage (1.8 V). That is, no current flows when the drain voltage is lower than the gate threshold voltage, which is why the device has forward drain turn-on characteristics that are similar to the gate threshold characteristics. As the drain voltage becomes higher than the forward turn-on voltage of the p-GaN/AlGaN/GaN heterojunction diode, the current can flow from both the p-GaN drain and ohmic drain regions to the source electrode. In the reverse region, when the drain voltage is negative, the p-GaN drain region is reverse-biased and it further depletes the channel, blocking the current flow from the drain. Therefore, the device exhibits reverse blocking characteristics. The electron concentration distributions under forward and reverse modes are compared in Figure 5a,b, respectively. The electron channel exists under the p-GaN drain region in the forward mode that is shown in Figure 5a, where both gate and drain voltages were +5 V. On the other hand, the channel under the p-GaN drain region was depleted in the reverse mode that is shown in Figure 5b where the gate and drain voltages were +5 V and −5 V, respectively.

## 3. Fabrication

### 3.1. Device Structure and Fabrication

**Figure 6:**
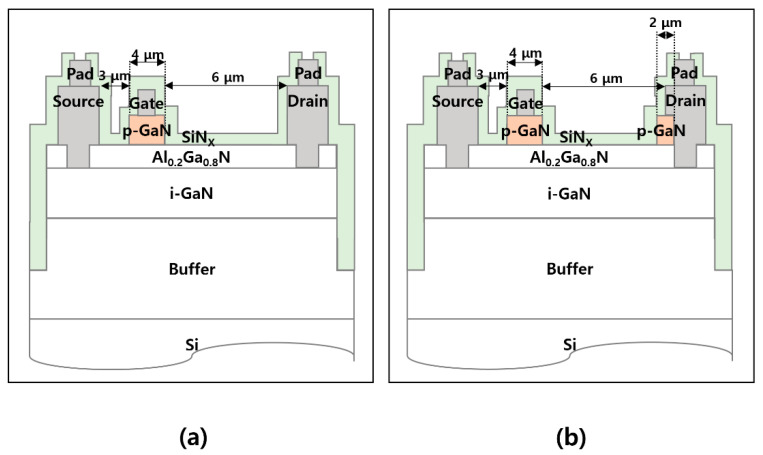
Cross-sectional schematics of (**a**) fabricated p-GaN gate AlGaN/GaN HFET and (**b**) unidirectional HFET.

Two device structures were fabricated to validate the proposed concept, as follows. Figure 6a,b shows the cross-sectional schematics of the conventional p-GaN gate AlGaN/GaN HFET and the unidirectional HFET, respectively. The epitaxial structure consisted of a 70 nm p-GaN layer, a 15 nm Al_0.2_Ga_0.8_N barrier layer, a 320 nm GaN layer, and a 3.6 μm buffer layer grown on a Si (111) substrate. After solvent and acid cleaning of the surface, the p-GaN layer was etched while using a two-step etching process, during which the gate and p-GaN drain regions were covered by photoresist. First, the p-GaN layer was partially etched by a low-damage plasma etching process using Cl_2_/BCl_3_-based inductively coupled plasma reactive ion etching (ICP-RIE) with an etch depth target of 45 nm. A source RF power of 250 W, a bias RF power of 5 W, a gas flow rate of Cl_2_/BCl_3_ = 18/2 sccm, and a chamber pressure of 5 mTorr were used, which resulted in an etch rate of ~1 Å/s. Subsequently, the remaining p-GaN layer was etched by a selective etching process using Cl_2_/N_2_/O_2_-based ICP-RIE to minimize the plasma-induced damage on the surface. A source RF power of 2000 W, a bias RF power of 25 W, a gas flow rate of Cl_2_/N_2_/O_2_ = 40/10/2 sccm, and a chamber pressure of 20 mTorr were used with a chuck temperature of 60 °C [20]. The selectivity between p-GaN and AlGaN was approximately 50:1 with a p-GaN etch rate of 3.6 Å/s. After the p-GaN layer was completely removed, the oxidized AlGaN surface was treated for 30 s using a buffered oxide etchant (30:1). Subsequently, damage recovery annealing was performed at 500 °C for 5 min. in an N_2_ ambient. The ohmic contact region was etched down to the GaN channel layer while using the low-damage BCl_3_/Cl_2_-based ICP-RIE with an etch depth of 15 nm, after which an additional photolithography process defined the ohmic metallization area with an overhang structure. The overhang region was extended to the p-GaN drain region for the unidirectional device, as shown in Figure 6b. A Ti/Al/TiN (=30/100/20 nm) metal stack was used for the Au-free ohmic contact, which was annealed at 550 °C for 1 min. in N_2_ ambient. The transfer contact resistance was 0.56 Ω∙mm. MESA isolation was then carried out using the BCl_3_/Cl_2_-based RIE with an etch depth of 450 nm. A forward power of 100 W, a gas flow rate of Cl_2_/BCl_3_ = 18/6 sccm, and a chamber pressure of 75 mTorr were used for the RIE process. Subsequently, a 170-nm TiN film was sputtered for the gate and pad electrode regions. The surface was passivated with a 180-nm SiNx film using ICP chemical vapor deposition (ICP-CVD). A RF power of 200 W, a gas flow rate of SiH_4_(5%)/N_2_/NH_3_ = 25/400/12 sccm, and a chamber pressure of 2000 mTorr were used with a chuck temperature of 350 °C. Finally, SF_6_-based ICP-RIE was used to open the probe contact region. Notably, the unidirectional device does not require an additional process step. The source-to-drain distance, p-GaN length for the gate region, and gate-to-drain distance were 3, 4, and 6 μm, respectively, where the gate metal length was 2 μm, and the ohmic overhang extension was 1 μm. The length of the p-GaN drain region was 2 μm in the unidirectional device.

### 3.2. Device Characteristics

**Figure 7:**
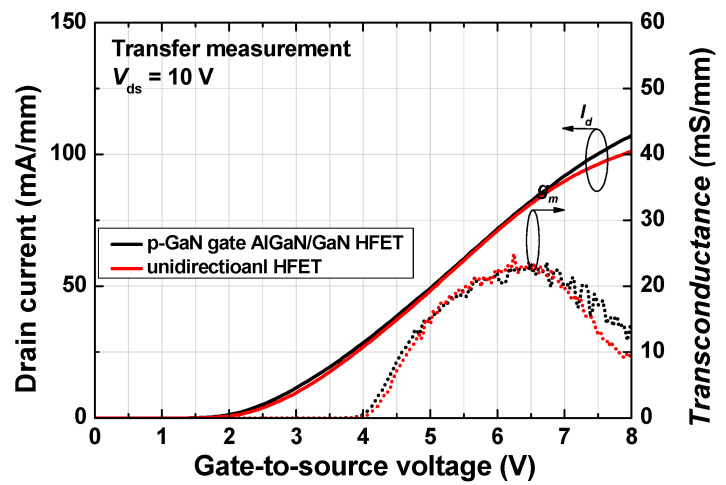
Transfer current–voltage characteristics of fabricated p-GaN gate AlGaN/GaN HFET (black line) and unidirectional HFET (red line).

Figure 7 shows the transfer current–voltage characteristics of the fabricated p-GaN gate AlGaN/GaN HFET (black lines) and unidirectional device (red lines) that were measured at a drain voltage of 10 V. No significant difference was observed between the two devices, in which the gate threshold voltage was ~2 V at 1 mA/mm.

**Figure 8:**
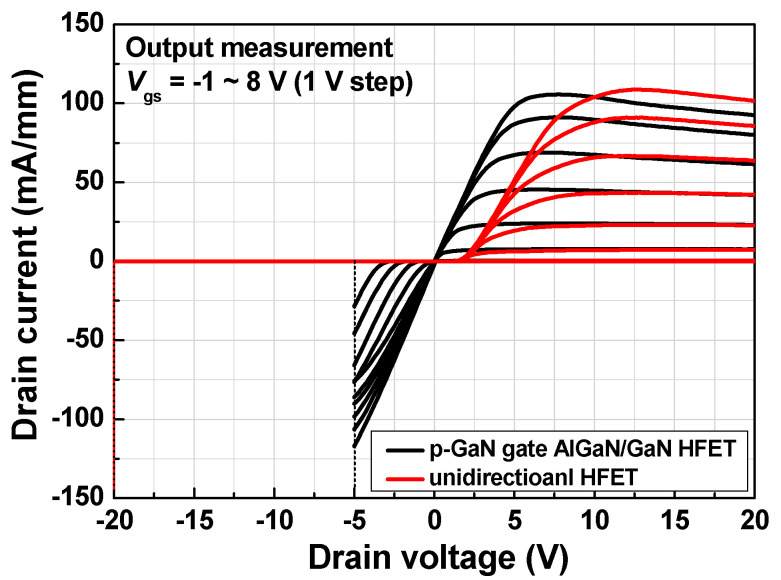
Output current–voltage characteristics of fabricated p-GaN gate AlGaN/GaN HFET (black line) and unidirectional HFET (red line).

Figure 8 shows the forward and reverse output current–voltage characteristics. The p-GaN gate AlGaN/GaN HFET (black lines) exhibited bidirectional characteristics, whereas the proposed device exhibited unidirectional characteristics (red lines). The forward drain turn-on voltage for the unidirectional device was ~2 V, which is the same as the gate threshold voltage, as discussed previously. A potential drawback of the proposed device is the forward drain turn-on characteristic. However, the overall device unit would have similar forward turn-on characteristics when an additional reverse blocking device is added to achieve the unidirectional characteristics. It is suggested that the p-GaN drain region be etched partially and/or a different metal contact be used for the p-GaN drain region in order to reduce the forward drain turn-on voltage.

## 4. Conclusions

A unidirectional p-GaN/AlGaN/GaN HFET was proposed to implement a normally-off, unidirectional operation, which was validated by both simulation and device demonstration. A p-GaN drain electrode was embedded in front of the ohmic drain electrode, in which they were electrically shorted to each other. The p-GaN drain region acted as a gate in the forward mode and as a reverse-biased rectifier in the reverse mode, which resulted in reverse blocking characteristics. The proposed device would be a cost-effective solution for achieving unidirectional operation, because it requires no separate reverse blocking device or an additional process step. The fabricated device exhibited a threshold voltage of ~2 V, a maximum current density of ~100 mA/mm, and a drain forward turn-on voltage of ~2 V. It is suggested that the drain turn-on voltage in the forward operation mode can be further reduced by the process engineering for the p-GaN drain contact.
